# BLOC-3 Mutated in Hermansky-Pudlak Syndrome Is a Rab32/38 Guanine Nucleotide Exchange Factor

**DOI:** 10.1016/j.cub.2012.09.020

**Published:** 2012-11-20

**Authors:** Andreas Gerondopoulos, Lars Langemeyer, Jin-Rui Liang, Andrea Linford, Francis A. Barr

**Affiliations:** 1 Department of Biochemistry, University of Oxford, South Parks Road, Oxford OX1 3QU, UK; 2 Wellcome Trust PhD Programme in Cellular and Molecular Physiology, Physiological Laboratory, Institute of Translational Medicine, University of Liverpool, Crown Street, Liverpool L69 3BX, UK

## Abstract

Hermansky-Pudlak syndrome (HPS) is a human disease characterized by partial loss of pigmentation and impaired blood clotting [1–3]. These symptoms are caused by defects in the biogenesis of melanosomes and platelet dense granules, often referred to as lysosome-related organelles [2]. Genes mutated in HPS encode subunits of the biogenesis of lysosome-related organelles complexes (BLOCs). BLOC-1 and BLOC-2, together with the AP-3 clathrin adaptor complex, act at early endosomes to sort components required for melanin formation and melanosome biogenesis away from the degradative lysosomal pathway toward early stage melanosomes [4–6]. However the molecular functions of the Hps1-Hps4 complex BLOC-3 remain mysterious [7–9]. Like other trafficking pathways, melanosome biogenesis and transport of enzymes involved in pigmentation involves specific Rab GTPases, in this instance Rab32 and Rab38 [10–12]. We now demonstrate that BLOC-3 is a Rab32 and Rab38 guanine nucleotide exchange factor (GEF). Silencing of the BLOC-3 subunits Hps1 and Hps4 results in the mislocalization of Rab32 and Rab38 and reduction in pigmentation. In addition, we show that BLOC-3 can promote specific membrane recruitment of Rab32/38. BLOC-3 therefore defines a novel Rab GEF family with a specific function in the biogenesis of lysosome-related organelles.

## Graphical abstract

**Graphical Abstract:**

**Figure:**
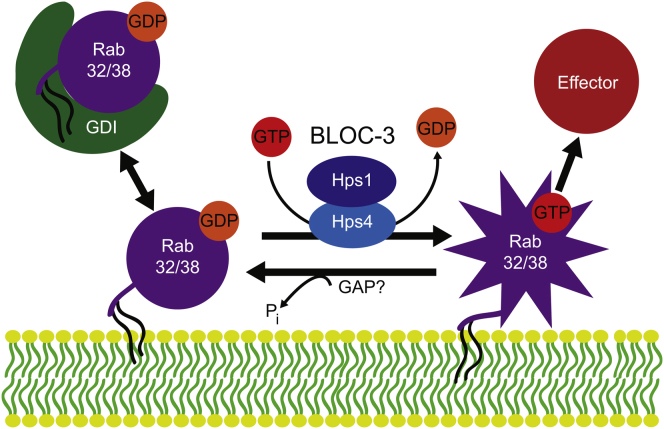

**Highlights:**

► The Hps1-Hps4 complex (BLOC-3) is a guanine nucleotide exchange factor (GEF) ► Purified BLOC-3 has specific GEF activity toward Rab32 and Rab38 ► BLOC-3 can promote recruitment of Rab32 and Rab38 to membranes ► BLOC-3 and its target Rabs act in the biogenesis of melanosomes

## Results and Discussion

### BLOC-3 Is a GEF for Rab32 and 38

**Figure 1:**
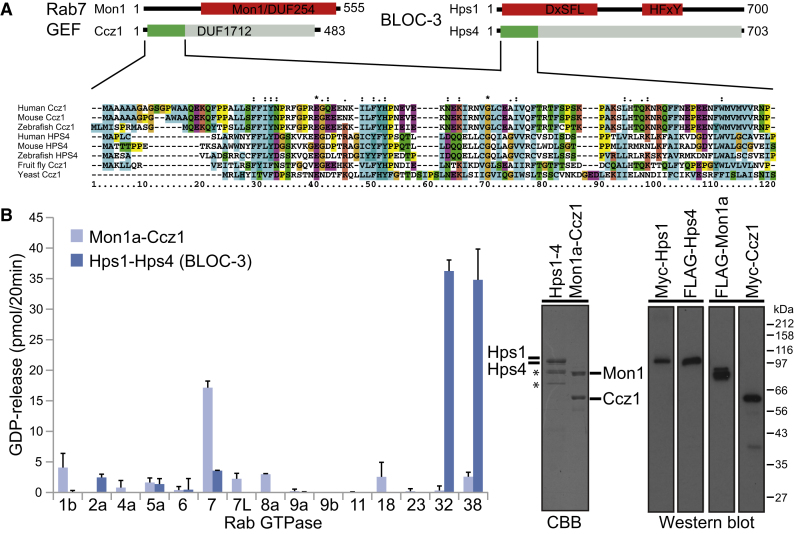
Hps1-Hps4 and Mon1a-Ccz1 Are Specific Rab GEFs (A) The putative Rab7 GEF is thought to be a heterodimer comprised of Mon1a and Ccz1 subunits. Each subunit is defined by a conserved domain of unknown function (DUF), as indicated schematically in the figure. BLOC-3 is a heterodimer comprised of Hps1 and Hps4 subunits. Sequence analysis indicates that Hps4 has 32% similarity throughout its entire length to the DUF1712 longin domain region of Ccz1. This similarity is most pronounced in the amino-terminal region shaded darker green and shown in the sequence alignment. Yeast Ccz1 is highly divergent and shows only limited similarity to the higher eukaryote proteins. Hps1 has 38% similarity to the DUF254 region of Mon1a, colored red in the figure. However, in Hps1 this domain is split into two sequence blocks containing the conserved motifs DxSFL and HFxY, respectively. (B) BLOC-3 and Mon1-Ccz1 complexes were used for GEF assays toward a representative group of Rab GTPases. Error bars indicate the standard deviation of the mean. The GEF complexes were analyzed by mass spectrometry and western blotting to exclude the presence of other proteins with possible Rab GEF activity. Protein gels were stained with colloidal Coomassie brilliant blue stain (CBB). Asterisks mark contaminating heat shock proteins in the Hsp1-Hps4 complexes.

Mutations in components of the Rab prenylation machinery required to link Rabs to membranes result in pigmentation defects due to altered platelet and melanosome formation [13]. Moreover, mutations in rat Rab38 cause pigmentation defects and other changes similar to those found in HPS [10], and Rab38 and the closely related Rab32 are important for trafficking of enzymes, such as Tyrp1, involved in pigmentation [11, 12]. However, it is not known how these Rabs become activated during melanosome biogenesis. This activation step requires the inactive Rab to release GDP and bind GTP, a process triggered by a specific guanine nucleotide exchange factor (GEF) [14]. Bioinformatic analysis of one of the genes mutated in Hermansky-Pudlak syndrome, Hps4, indicated the amino-terminal 120 amino acids were homologous to the equivalent region of the Ccz1 subunit of the yeast Ypt7 GEF (Figure 1A) [15–17]. In addition, similarities were noted between Hps1 and the Mon1 subunit of the Ypt7 GEF (Figure 1A) [18]. Intriguingly, given these similarities between Hps1-Hps4 and Mon1-Ccz1, Rab32 and Rab38 fall into the same subcategory of Rabs as Rab7. To test if BLOC-3 has Rab GEF activity, recombinant Hps1-Hps4 complexes and Rab7 GEF complexes were produced (see Figures S1A and S1B available online). Due to the low sequence similarity of human and the characterized yeast Mon1-Ccz1 complex, the human protein complex was first isolated and the subunit composition confirmed by mass spectrometry (Figure S1C). These complexes were then tested for GEF activity toward a panel of Rab7 subfamily Rabs, as well as a selection of other Rabs (Figure 1B). This analysis showed that the BLOC-3 complex but not the subunits alone had GEF activity toward Rab32 and the closely related Rab38, but little or no activity to the other Rabs tested (Figures 1B and S1D). Bacterially expressed BLOC-3 also showed activity to Rab32 and Rab38 but not Rab7 or Rab7-like (Figure S1E), eliminating the possibility that a eukaryotic cell contaminant was responsible for the Rab32/38 GEF activity. By contrast, human Mon1a-Ccz1 had activity toward Rab7 but little or no activity toward Rab32, Rab38, or other Rabs tested (Figure 1B). These findings supported the idea that BLOC-3 and Mon1a-Ccz1 are specific Rab GEF complexes for Rab32/38 and Rab7, respectively.

### BLOC-3 Promotes Membrane Localization of Rab32 and 38

**Figure 2:**
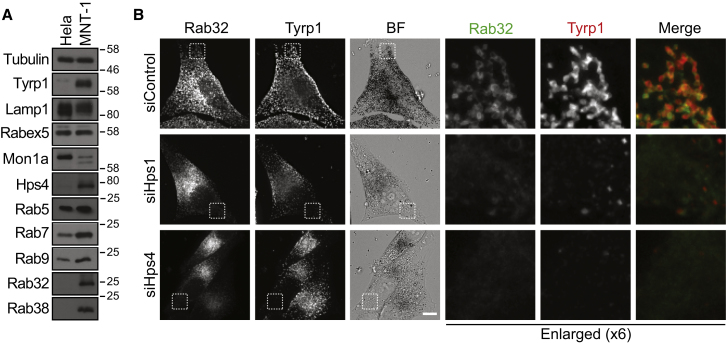
BLOC-3 Is Required for Rab32 and Rab38 Localization (A) Equal amounts of HeLa S3 and MNT-1 cell extract were blotted for endocytic Rab GTPases and their GEF regulators. Tyrp1 and LAMP1 were used as markers for melanosomes and lysosomes, respectively. (B) MNT-1 cells treated with control, Hps1, or Hps4 siRNA duplexes for 6 days were washed in growth medium, replated and transfected with GFP-Rab32 for 48 hr, and then PFA-glutaraldehyde fixed. Cells were stained for Tyrp1 and DAPI to detect DNA. A bright-field image (BF) shows the pigmented mature melanosome structures. Scale bar represents 10 μm in nonenlarged panels.

If BLOC-3, the Hps1-Hps4 complex, acts as a specific GEF for Rab32/38 in vivo, then the recruitment of Rab32/38 to membranes, the localization of melanosomal proteins, and the formation of melanosomes should require the activity of this complex. All of these properties were therefore tested using MNT-1 cells, which produce melanosomes. Western blot analysis of HeLa and MNT-1 cells revealed that the BLOC-3 subunit Hps4 is enriched in MNT-1 cells, like its target GTPases Rab32 and Rab38, and the tyrosinase-related protein (Tyrp1), a marker for melanin biogenesis (Figure 2A). Rabs and regulators required for early endocytic trafficking and lysosomal sorting were present in both cell lines, although MNT-1 cells have lower levels of the Rab7 GEF subunit Mon1a (Figure 2A). Rab32 was present on small ring-like structures in control cells that overlapped with Tyrp1 (Figure 2B), characteristic of endosomal and melanosomal structures required for sorting of melanosome components [12]. When either the Hps1 or Hps4 subunits of BLOC-3 were depleted using different siRNA duplexes, Rab32 was lost from these ring-like membranes and became redistributed to a diffuse or finely punctate cytoplasmic pattern closer to the nucleus (Figures 2B and S2A). This was in agreement with the measurements showing that both subunits of BLOC-3 are required for GEF activity toward Rab32 or 38. Furthermore, Rab32 localizes to structures adjacent to those defined by the Hps4 subunit of its GEF (Figure S2B).

**Figure 3:**
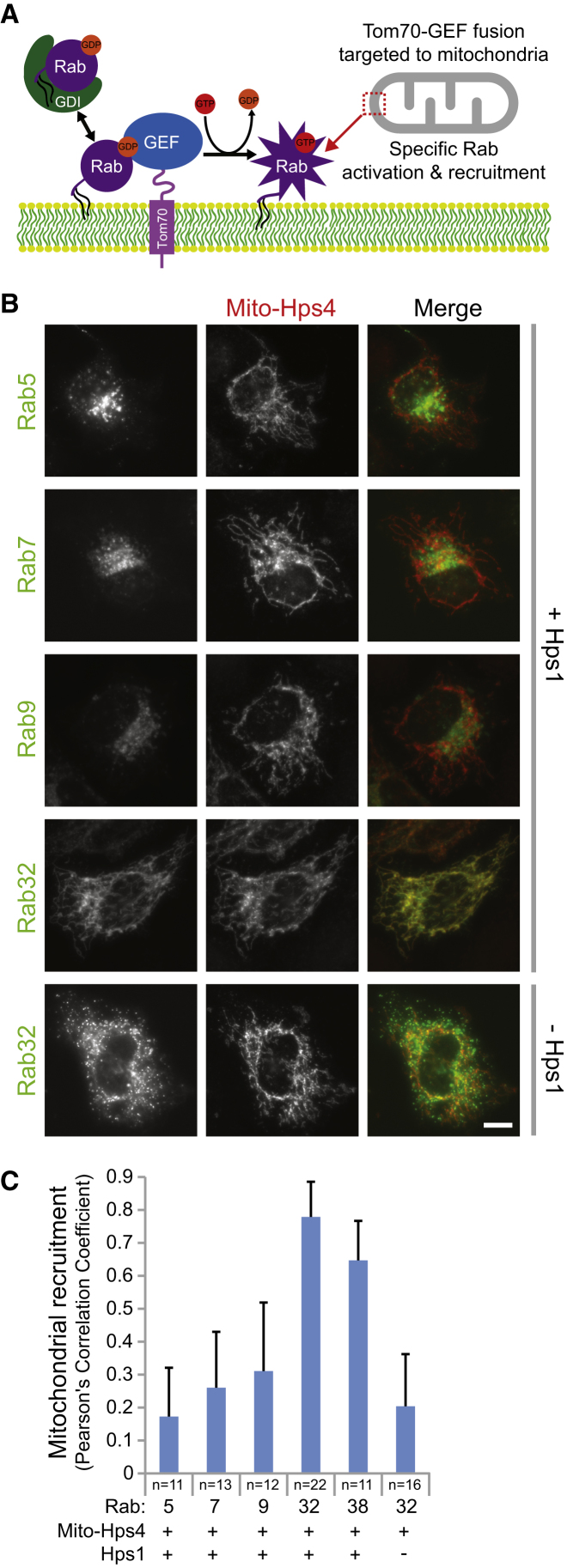
BLOC-3 Is Sufficient to Determine Rab32 Localization (A) Schematic outlining the basis of the mitochondrial targeting assay for Rab GEF activity (MitoGEF assay system). (B) HeLa cells were cotransfected for 20 hr with eGFP-Rabs and the Tom70-FLAG-Hps4 fusion (Mito-Hps4), in the presence and absence of Myc-Hps1. The cells were PFA fixed and then stained with Hps4 antibodies; Rabs were visualized using GFP fluorescence. The scale bar represents 10 μm. (C) The extent of mitochondrial recruitment of the different GTPases was measured by calculating Pearson’s correlation coefficient for the GFP and Mito-Hps4 signals. This is plotted in the graph, and error bars show standard deviation from the mean for the number of samples indicated in the figure.

To provide further support for the idea that BLOC-3 is important for determining the localization of Rab32, the Hps1-Hps4 complex was relocated to the surface of a heterologous membrane, the mitochondria (Figure 3A). This resulted in the recruitment of Rab32 to the surface of the mitochondria only when both Hps1 and Hps4 were expressed (Figures 3B, 3C, and S3A). Importantly, in this system Hps1 and Hps4 formed a complex at the surface of mitochondria (Figures S3A and S3B), consistent with the idea that the BLOC-3 complex and not the subunits alone have GEF activity. Other Rabs were tested in this assay but did not show mitochondrial targeting (Figures 3B and 3C), supporting the conclusion that this reflected a specific activity of BLOC-3 on Rab32 and Rab38. Together, these data showed that BLOC-3 activity is required for Rab32 and Rab38 activation and is an important determinant of its localization to premelanosomal membrane structures.

### BLOC-3 Plays a Role in Melanosome Formation

**Figure 4:**
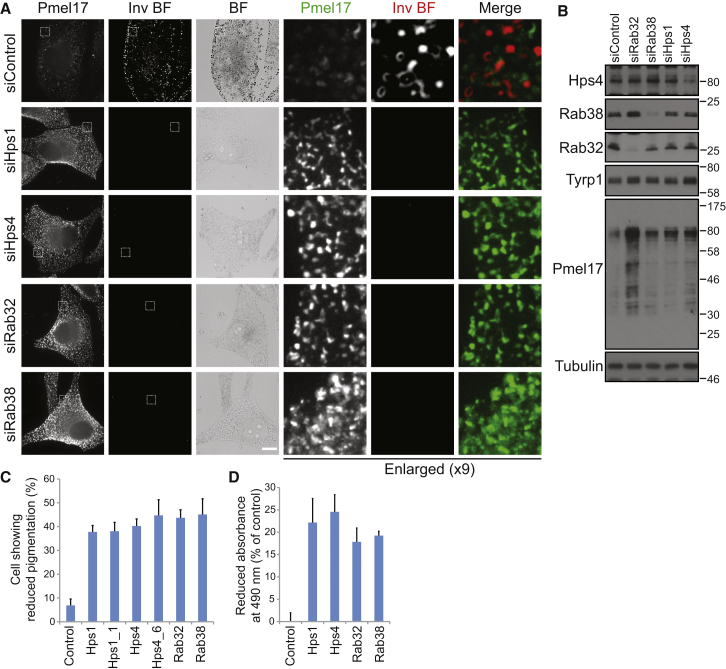
BLOC-3 Is Required for Melanosome Formation from Premelanosomes (A) MNT-1 cells treated with control, Hps1, Hps4, Rab32, and Rab38 siRNA duplexes for 8 days were PFA-glutaraldehyde fixed and then stained for PMEL. A bright-field image (BF) was taken and inverted to more clearly show the dark melanosomes (Inv BF). Scale bar represents 10 μm in nonenlarged panels. (B) Equivalent samples were western blotted to confirm depletion of the various target proteins and for melanosome pathway markers Tyrp1 and PMEL. Tubulin was used as a loading control. (C) The number of cells showing reduced or altered (aggregated) melanosomes was counted and is plotted in the graph; error bars reflect the standard deviation from the mean (n = 3). (D) Direct measurements of pigment were carried out using cell pellet-associated absorbance at 490 nm. A representative example from three independent experiments is shown. Error bars reflect duplicate samples.

Finally, the role of BLOC-3 in trafficking of melanosomal cargo and production of melanosomes was tested. Previous studies have shown that cells from mice lacking Rab32/38 display deficiencies in the formation of mature melanosomes and accumulate early stage melanosomes [11, 12]. If BLOC-3 is required for Rab32/38 activation, then cells depleted of Hps1, Hps4, and Rab32/38 should show a similar accumulation of premelanosomal structures and reduced numbers of mature heavily pigmented melanosomes. For this purpose the premelanosome marker PMEL was used. PMEL is a transmembrane glycoprotein proteolytically cleaved following exit from the TGN as part of the normal melanosome biogenesis pathway [19–21]. Precursor forms of PMEL are incorporated into insoluble fibrils and cleaved to 35–45 kDa fragments that become buried by melanin within melanosomes [22]. In control cells, PMEL localizes to a punctate pattern clearly discrete from the mature melanosomes seen in the inverted bright-field image (Figure 4). Depletion of Rab32/38, Hps1, or Hps4 resulted in a strongly increased PMEL signal (Figure 4A). Western blotting showed this was due to increased levels of an 80 kDa nonprocessed form of PMEL in the cells (Figure 4B), suggesting that the proteolytic maturation of PMEL and its burial by melanin was blocked. Furthermore, there was a decrease in densely pigmented melanosomes in nearly 50% of cells (Figures 4A and 4C) and a 20%–30% reduction in pigment production (Figure 4D). This was especially obvious in bright-field images where the dark, round melanosomes are clearly reduced or show altered distribution, sometimes clustering together in large aggregates (Figure S4). BLOC-3 and its targets, Rab32 and Rab38, therefore play an important role in the control of pigment production and melanosome biogenesis in cultured MNT-1 cells.

### Conclusions

These findings provide insights into membrane trafficking pathways of great relevance for both normal cellular function and human diseases. Together the data demonstrate that Hps1-Hps4 (BLOC-3) and Mon1a-Ccz1 form specific Rab GEF complexes for Rab7 and Rab32/38, with discrete functions in the biogenesis of lysosome-related organelles and lysosomes, respectively. The characterization of BLOC-3 and Mon1-Ccz1 therefore defines a new family of Rab GEFs and supports the view that the longin domain is a signature for Rab GEF activity [14, 23]. Intriguingly, some SNAREs possess longin domains [17, 24], suggesting that these might directly link Rab regulation to vesicle tethering and fusion events in a manner not previously suspected.

Other unanswered questions relate to the mechanism by which BLOC-3 is recruited and triggers local activation of Rab32 and Rab38. One possibility is that this takes the form of a Rab cascade, where an upstream Rab promotes the recruitment of the exchange factor for downstream Rabs in the pathway [25]. Fitting with this idea, BLOC-3 has been shown to bind to the activated GTP form of Rab9 [26]. Intriguingly, the Rab32 and Rab38 GTPase-activating protein RUTBC1 is also a Rab9 effector [27]. These observations suggest that Rab9 may play a previously unappreciated role in the formation of lysosome-related organelles by coordinating the activation and inactivation of Rab32 and Rab38. In turn, Rab32 and Rab38 may then feed back to regulate yet further Rabs, since the VARP exchange factor for Rab21 is a Rab32/38-binding protein [28]. Further work will be required to shed light on the currently murky picture of how such a complex cascade might function.

## Supplemental Information

Document S1. Supplemental Experimental Procedures and Four Figures

Table S1. siRNA Sequences Used in This Study, Related to Supplemental Experimental Procedures
