# Supplementary material for: BLOC-3 Mutated in Hermansky-Pudlak Syndrome Is a Rab32/38 Guanine Nucleotide Exchange Factor
Source: Curr Biol. 2012 Nov 20;22(22):2135–9. doi: 10.1016/j.cub.2012.09.020 (PMC3502862; doi:10.1016/j.cub.2012.09.020)
Supplement: Document S1. Supplemental Experimental Procedures and Four Figures [file mmc1.pdf]

Current Biology, Volume *22*

Supplemental Information

BLOC-3 Mutated in Hermansky-Pudlak Syndrome Is a Rab32/38 Guanine Nucleotide Exchange Factor

Andreas Gerondopoulos, Lars Langemeyer, Jin-Rui Liang, Andrea Linford, and Francis A. Barr

Supplemental Experimental Procedures

Reagents and Antibodies

General laboratory chemicals were obtained from Sigma-Aldrich and Fisher Scientific. Antibodies were raised against recombinant hexahistidine-tagged Mon1a and Hps4, and affinity purified on the respective antigen coupled to Affigel-15 (Biorad). Commercially available antibodies were used to α-tubulin (mouse DM1A; Sigma), Myc-epitope (mouse clone 9E10; Sigma), FLAG-epitope (mouse monoclonal M2; Sigma), EEA1 (rabbit #2411; Cell Signaling Technology), LAMP1 (mouse 1D4B; Developmental Studies Hybridoma Bank, University of Iowa), Rab32 (rabbit SAB4200086; Sigma), Rab38 (rabbit #9688; Cell Signaling Technology), Rabex5 (rabbit N-terminal antibody; Sigma), Rab5 (rabbit clone C8B1; Cell Signaling Technology), Rab7 (rabbit clone D95F2; Cell Signaling Technology), Rab9 (rabbit clone D52G8; Cell Signaling), PMEL (mouse clone HMB45; Abcam), Tyrp1 (mouse clone Ta99; Novus Biologicals). Secondary antibodies raised in donkey to mouse, rabbit, sheep/goat, and human conjugated to HRP, Alexa-488, Alexa-555, Alexa-568, and Alexa-647 were obtained from Molecular Probes and Jackson ImmunoResearch Labs.

Molecular Biology and Rab Protein Expression

The libraries of Rab GTPases and siRNA duplexes, and detailed methods for Rab protein expression have been described previously [1, 2]. Human Mon1, Ccz1 (C7ORF28), Hps1, and Hps4 were amplified by PCR from cDNA. Duplexes for BLOC-3 and Mon1a-Ccz1 siRNA were obtained from Qiagen or Dharmacon

and are listed in the Table S1. A GL2 duplex was used as a control in all siRNA experiments. Mammalian expression constructs were made using pcDNA4/TO and pcDNA5/FRT/TO vectors (Invitrogen). Mitochondrially targeted BLOC-3 was created in pcDNA5 by fusing the mitochondrial outer membrane targeting sequence of yeast Tom70p and 3 copies of the FLAG epitope sequence to the 5-prime end of the Hps4 cDNA. Bacterial expression constructs were made using pQE32 (Qiagen) and pFAT2, encoding the His_6-tag and His_6-glutathione-S-transferase, respectively. For co-expression of Hps1 and Hps4 in bacteria they were inserted into cloning sites 1 and 2, respectively, of pETDuet-1 (Novagen), and expressed in BL21(DE3).

Cell Culture

HeLa, and HEK293 cells were cultured in DME (Dulbecco's modified Eagle medium) containing 10% bovine calf serum (Invitrogen) at 37°C and 5% CO_2. For plasmid and siRNA transfection of HeLa or HEK293T cells Mirus LT1 (Mirus Bio LLC) and Oligofectamine (Invitrogen), respectively, were used according to the manufacturers instructions. MNT-1 cells were cultured in 70% DME and 10% AIM-V medium (Invitrogen), supplemented with 20% bovine calf serum, 1x non-essential amino acids (Invitrogen), and 1 mM sodium pyruvate at 37°C and 5% CO_2. For plasmid and siRNA transfection of MNT-1 cells Mirus LT1 (Mirus Bio LLC) and siQuest (Qiagen), respectively, were used according to the manufacturers instructions.

Purification of GEF Complexes

FLAG and Myc-tagged forms of Mon1 and Ccz1, or Hps1 and Hps4 were transiently expressed in 8x 15 cm dishes of 70% confluent HEK293T cells. For this purpose 800 µl OptiMEM (Invitrogen) was mixed with 24 µl Mirus LT1, and after 5 minutes 6 µg of each plasmid DNA added. After 25 minutes this transfection mix was added to the cells. After 40 hours growth the cell pellet was lysed for 20 minutes on ice in 5 ml cell lysis buffer (50 mM Tris-HCl pH 7.4, 1 mM EDTA, 150 mM NaCl, 0.5 % [vol/vol] Triton-X 100, protease inhibitors cocktail). Cell extracts were split into 1 ml aliquots and clarified by centrifugation at 20,000x g in an Eppendorf 5417R microfuge for 20 minutes. The FLAG-tagged proteins were isolated from the clarified cell lysate using 100 µl anti-FLAG M2 affinity gel (Sigma) for 4 hours at 4°C. The beads were washed ten times in 1 ml cell lysis buffer, ten times in 1 ml high salt buffer (50mM Tris-HCl pH 7.4, 500 mM NaCl), ten times in TBS, and finally the proteins were eluted with 100 µl 200 µg/ml FLAG-peptide in TBS containing 2 mM dithiothreitol. The eluted proteins were analysed on 7.5-10% SDS-PAGE gels stained with Coomassie brilliant blue, and concentrations estimated by comparison to a series of bovine serum albumin standards in the range 0.1 to 1 mg. The peak fractions were snap frozen in liquid nitrogen for storage at -80°C without dialysis.

For bacterial expression, a 4 litre culture of BL21(DE3) transformed with pETDuet-1 containing Hps1 and Hps4 as described was grown to an OD600 of 0.6 then either induced overnight with 0.2 mM IPTG at 25°C or for 3 hours with 1 mM IPTG at 37°C. The cells were then harvested by centrifugation and purified

using nickel-agarose using the same procedure described for Rab GTPases [1, 2]. The eluted proteins were analysed on 7.5-10% SDS-PAGE gels stained with Coomassie brilliant blue, and concentrations estimated by comparison to a series of bovine serum albumin standards in the range 0.1 to 1 mg. The peak fractions were snap frozen in liquid nitrogen for storage at -80°C without dialysis.

Nucleotide Binding and GEF Assays

GEF assays were carried out as described previously [2]. Nucleotide loading was carried out as follows: 10 µg GST-tagged Rab was incubated in 50 mM HEPES-NaOH pH 6.8, 0.1 mg/ml BSA, 125 µM EDTA, 10 µM Mg-GDP, and 5 µCi [^{3}H]-GDP (10 mCi/ml; 5000 Ci/mmol) in a total volume of 200 µl for 20 min at 4°C. For standard GDP-releasing GEF assays 100 µl of the loading reaction was mixed with 10 µl 10 mM Mg-GTP, 10-100 nM GEF protein to be tested or a buffer control, and adjusted with assay buffer to 120 µl final volume. The GEF reaction occurred for 20 minutes at 30°C. After this, 2.5 µl were taken for a specific activity measurement, the remainder was split into two tubes, then incubated with 500 µl ice-cold assay buffer containing 1 mM $MgCl_2$, and 20 µl packed glutathione-sepharose for 60 minutes at 4°C. After washing 3 times with 500 µl ice-cold assay buffer the sepharose was transferred to a vial containing 4 ml scintillation fluid and counted. The amount of nucleotide exchange was calculated in pmoles GDP-released. For GTP-binding assays the following modifications were made: only unlabelled GDP was used in the loading reaction; in the GEF reaction 0.5 µl 10 mM GTP and 1 µCi [^{35}S]-GTPγS (10 mCi/ml; 5000

Ci/mmol) were used. The amount of nucleotide exchange was calculated in pmoles GTP-bound.

MitoGEF Assays

For MitoGEF immune fluorescence assays, HeLa cells were seeded on 1.5H glass coverslips at a density of 30,000 cells per well on a 12 well plate, and then left for 30 hours to adhere. The cells were transfected with 0.25 µg of mitochondrially targeted Tom70-3xFLAG-Hps4, Myc-Hps1, and eGFP-tagged Rab GTPases in combination using 1.5 µl Mirus LT1 in 50 µl OptiMEM. After 20 hours the cells were processed for microscopy using PFA fixation. For immune precipitations, a confluent 15 cm dish of HeLa cells was transfected with 8 µg of Tom70-3xFLAG-Hps4 and Myc-Hps1 in combination as described in the relevant figure using 24 µl Mirus LT1 and 800 µl OptiMEM. After 40 hours, cells were lysed in 1 ml cell lysis buffer and immune precipitations with FLAG antibodies carried out as described for GEF complexes.

MNT-1 Cell Lysis and Pigmentation Assays

MNT-1 cells treated as described in the figure legends were washed twice in 2 ml PBS, then removed from the dish by pipetting in 2 ml PBS containing 1 mM EDTA. The cells were harvested by centrifugation at 1000 g at 4°C in an Eppendorf 5417R microfuge for 5 minutes. The cell pellet was lysed for 20 minutes on ice in 1 ml cell lysis buffer (50 mM Tris-HCl pH 7.4, 1 mM EDTA, 150 mM NaCl, 0.5 % [vol/vol] Triton-X 100, protease inhibitors cocktail). Cell extracts

were clarified by centrifugation at 20,000 g in an Eppendorf 5417R microfuge for 30 minutes. For western blot analysis 10 µg of cell lysate used with the antibodies described in the figures. For pigmentation measurements the cell pellet containing the melanosomes was washed in 500 µl 1:1 ether ethanol, then air dried in a fume hood. This pellet was solubilised in 1 ml 2M NaOH, 20% DMSO by vigorous shaking in a tabletop mixer at 60°C for 3 hours. The absorbance was then measured at 490 nm in a spectrophotometer.

Mass Spectrometry

Protein samples for mass spectrometry were separated on 4-12% gradient NuPAGE gels, then stained using a colloidal Coomassie blue stain. Gel lanes were typically cut into 12 slices, and then digested with trypsin using published methods. The resulting tryptic peptide mixtures in 0.05% [vol/vol] trifluoracetic acid were then analyzed by online LC-MS/MS with a nanoAcquity UPLC (Waters) and Orbitrap XL ETD mass spectrometer (Thermo Scientific) fitted with a Proxeon nano-electrospray source. Peptides were loaded on to a 5cm x 180 µm BEH-C18 Symmetry trap column (Waters, part#186003514) in 0.1% formic acid at 15 µl/minute, and then resolved using a 25cm x 75 µm BEH-C18 column (Waters, part#186003815) in 99 to 37.5% acetonitrile in 0.1% [vol/vol] formic acid at a flow rate of 400 nl/minute. Mass spectra were acquired in the Orbitrap (R=30,000) and then MS/MS performed using collision ionization induced fragmentation (30 msec, 35% energy) on the top 5 ions not previously analysed in the linear quadrupole ion trap. Maxquant and Mascot (Matrix Science) were

then used to compile and search the raw data against the human IPI database [3].

Microscopy and Image Analysis

MNT-1 cells were grown on No. 1.5 glass coverslips (Fisher Scientific) and treated as described in the figure legends. For MitoGEF assays HeLa cells were grown as described in the methods section. For PFA fixation, coverslips were washed twice with 2 ml of PBS, and fixed with 2 ml of 3% [wt/vol] paraformaldehyde in PBS for 15 min. Coverslips were quenched with 2 ml of 50 mM NH_4Cl in PBS, and then incubated in a further 2 ml quench solution for 10 minutes. For PFA-glutaraldehyde fixation, coverslips were washed twice with 2 ml of PBS, and fixed with 2 ml of 0.1% [wt/vol] glutaraldehyde, 3% [wt/vol] paraformaldehyde in PBS for 15 min. Quenching was carried out using 0.5 mg/ml sodium borohydride in PBS for 3 times 5 minutes. For all fixation protocols, coverslips were washed three times in 2 ml PBS before permeabilization in 0.2% [vol/vol] Triton-X 100 for 5 minutes. Primary and secondary antibody staining was carried out in PBS for 60 minutes at room temperature. Affinity purified antibodies were used at 1 µg/ml while commercial antibodies were used as directed by the manufacturers. Fixed samples on glass slides were imaged using a 60 x 1.35 NA oil immersion objective on an Olympus BX61 upright microscope system with filter sets for DAPI, EGFP/Alexa Fluor 488, Alexa Fluor 555, Alexa Fluor 568, and Alexa Fluor 647 (Chroma Technology Corp.), a camera (CoolSNAP HQ2; Roper Industries), and imaging software (MetaMorph 7.5; Molecular Dynamics, Inc.).

Maximum intensity projection images of the fluorescent channels were cropped in NIH ImageJ and placed into Adobe Illustrator CS3 to produce the figures. Co-localisation analysis and calculation of Pearson's correlation coefficient was carried out using Volocity 5 (Perkin-Elmer).

Supplemental References

1. Yoshimura, S., Egerer, J., Fuchs, E., Haas, A.K., and Barr, F.A. (2007). Functional dissection of Rab GTPases involved in primary cilium formation. J Cell Biol *178*, 363-369.

2. Yoshimura, S., Gerondopoulos, A., Linford, A., Rigden, D.J., and Barr, F.A. (2010). Family-wide characterization of the DENN domain Rab GDP-GTP exchange factors. J Cell Biol *191*, 367-381.

3. Cox, J., and Mann, M. (2008). MaxQuant enables high peptide identification rates, individualized p.p.b.-range mass accuracies and proteome-wide protein quantification. Nat Biotechnol *26*, 1367-1372.

Figure S1. Gerondopoulos et al.

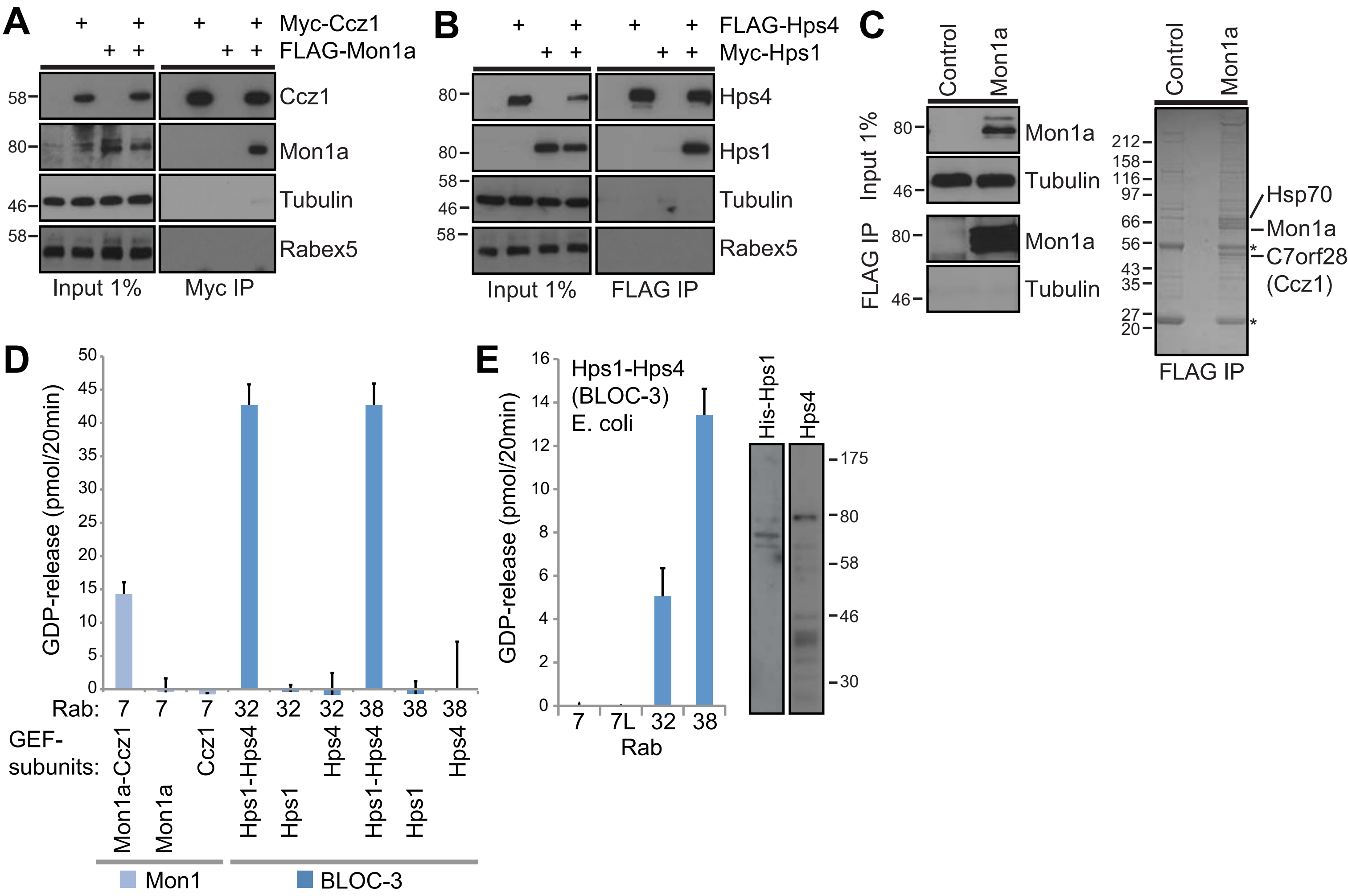

Figure S1. Production and Characterisation of Human Hps1-Hp4 and Mon1-Ccz1 Complexes

(A and B) HeLa cells were co-transfected for 24 hours with (A) Myc- and FLAG-tagged Mon1a and Ccz1, or (B) Hps1 and Hps4. The cells were then lysed and the complexes isolated by immune precipitation with FLAG or Myc antibodies as shown in the figure.

(C) Because of the low sequence similarity of yeast and the candidate human Ccz1, Mon1a complexes were isolated from human cells to confirm that endogenous human Ccz1 does interact with Mon1a. HeLa cells were transfected with a FLAG-tagged copy of Mon1a for 40 hours, then the cells lysed, and Mon1a immune-precipitated used FLAG-agarose. Immune complexes were washed, then analysed by SDS-PAGE, western blotting, and mass spectrometry.

(D) HeLa cells were transfected for 24 hours with Myc- and FLAG-tagged Mon1a and Ccz1, or Hps1 and Hps4 in combination. The cells were then lysed and the complexes isolated by immune precipitation with FLAG or Myc antibodies as shown in the figure. These GEF complexes or individual subunits complexes were also used for GEF assays towards a representative group of Rab GTPases. Error bars indicate the standard deviation of the mean.

(E) His-tagged Hps1-Hps4 complex expressed in and purified from bacteria was tested for GEF activity towards Rab7, 7-like, 32, and 38. Error bars indicate the standard deviation of the mean. Western blot analysis of the complex is shown to the right.

Figure S2. Gerondopoulos et al.

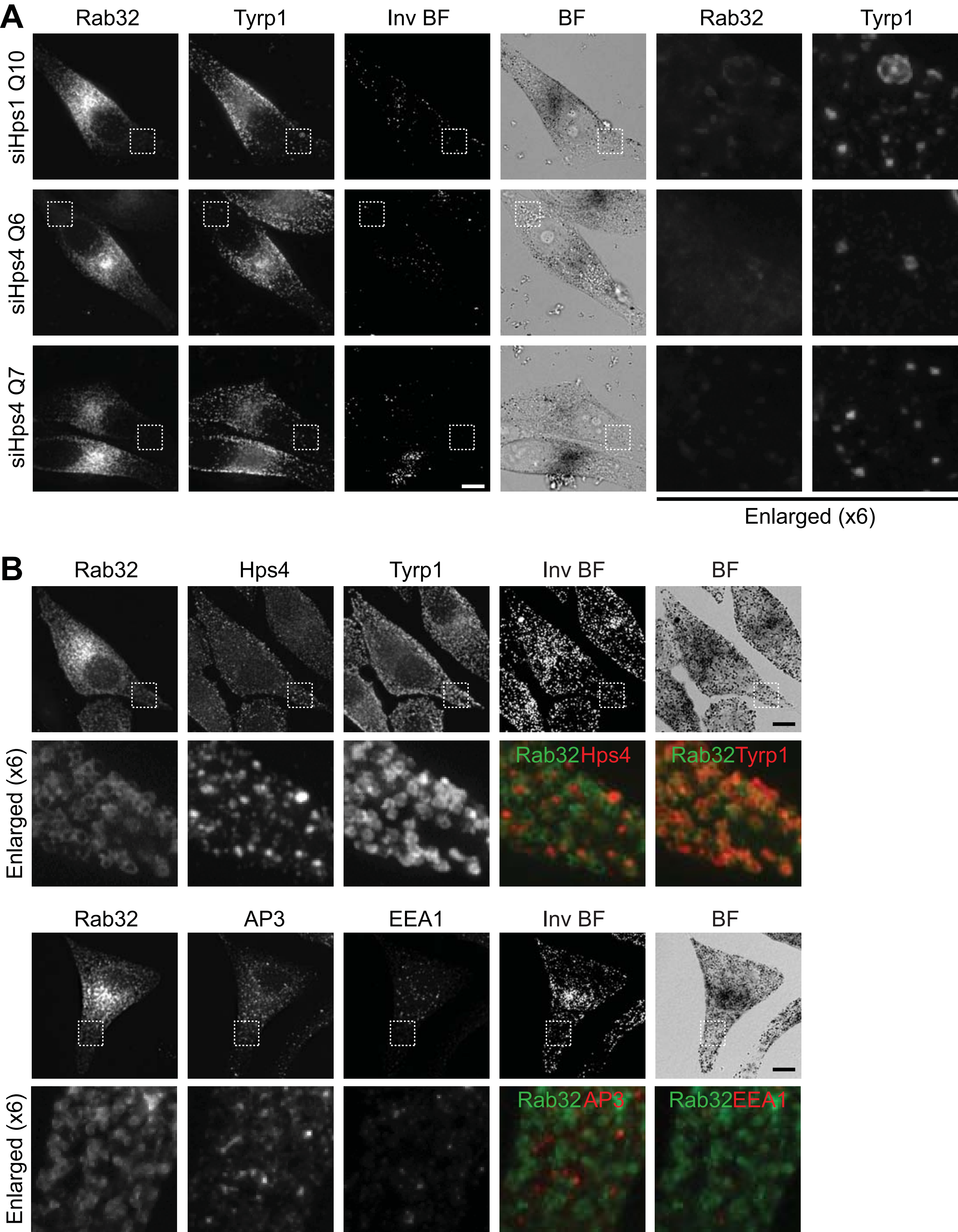

Figure S2. Rab32 Localisation Is Altered in Hps1 or Hps4 Depleted Cells

(A) MNT-1 cells treated with Hps1Q10, Hps4Q6 or Hps4Q7 siRNA duplexes for 6 days were washed in growth medium, re-plated and transfected with GFP-Rab32 for 48 hours, and then PFA-glutaraldehyde fixed. Cells were stained for Tyrp1 and DAPI to detect DNA. Scale bar is 10 µm in non-enlarged panels. Controls and other conditions are shown in Figure 2.

(B) MNT-1 cells transfected with eGFP-Rab32 for 48 hours were PFA fixed, and then stained with antibodies to AP3, EEA1, Hps4, and Tyrp1. Scale bar is 10 µm in non-enlarged panels. Rab32 shows overlap with Tyrp1 and less so with Hps4, but no overlap with AP3 or EEA1.

Figure S3. Gerondopoulos et al.

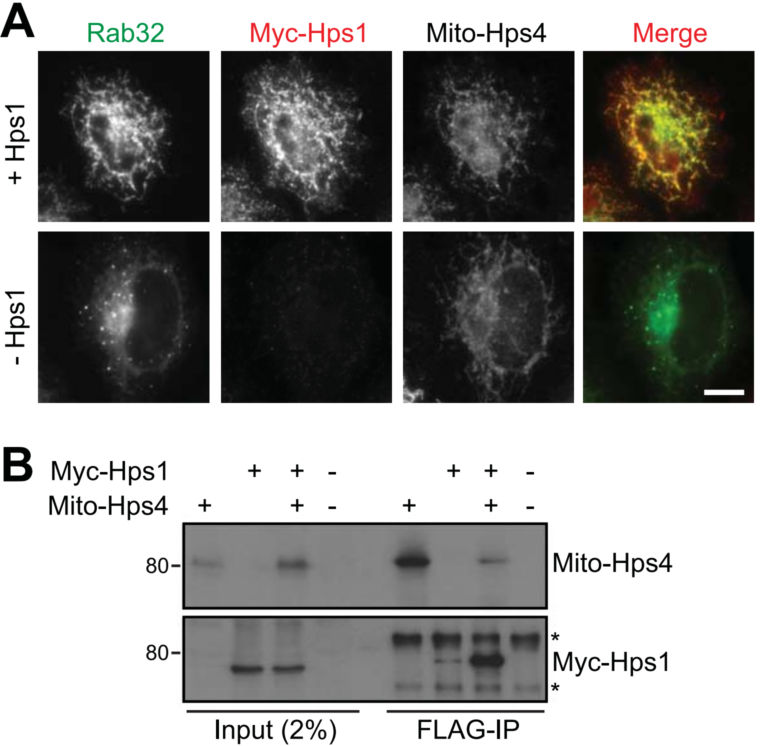

Figure S3. Mitochondrial Targeting of the Hps1-Hps4 Complex

(A) HeLa cells were co-transfected for 24 hours with eGFP-Rabs, the Tom70-FLAG-Hps4 fusion (Mito-Hps4) in the presence and absence of Hps1. The cells were PFA fixed and then stained with Hps4 and Myc-epitope antibodies, Rabs were visualised using GFP fluorescence. The scale bar is 10 µm.

(B) Alternatively, the cells were lysed and the Mito-Hps4 complexes isolated by immune precipitation with FLAG antibodies as western blotted shown in the figure. Asterisks mark non-specific cross-reactivity on the Myc-blot.

Figure S4. Gerondopoulos et al.

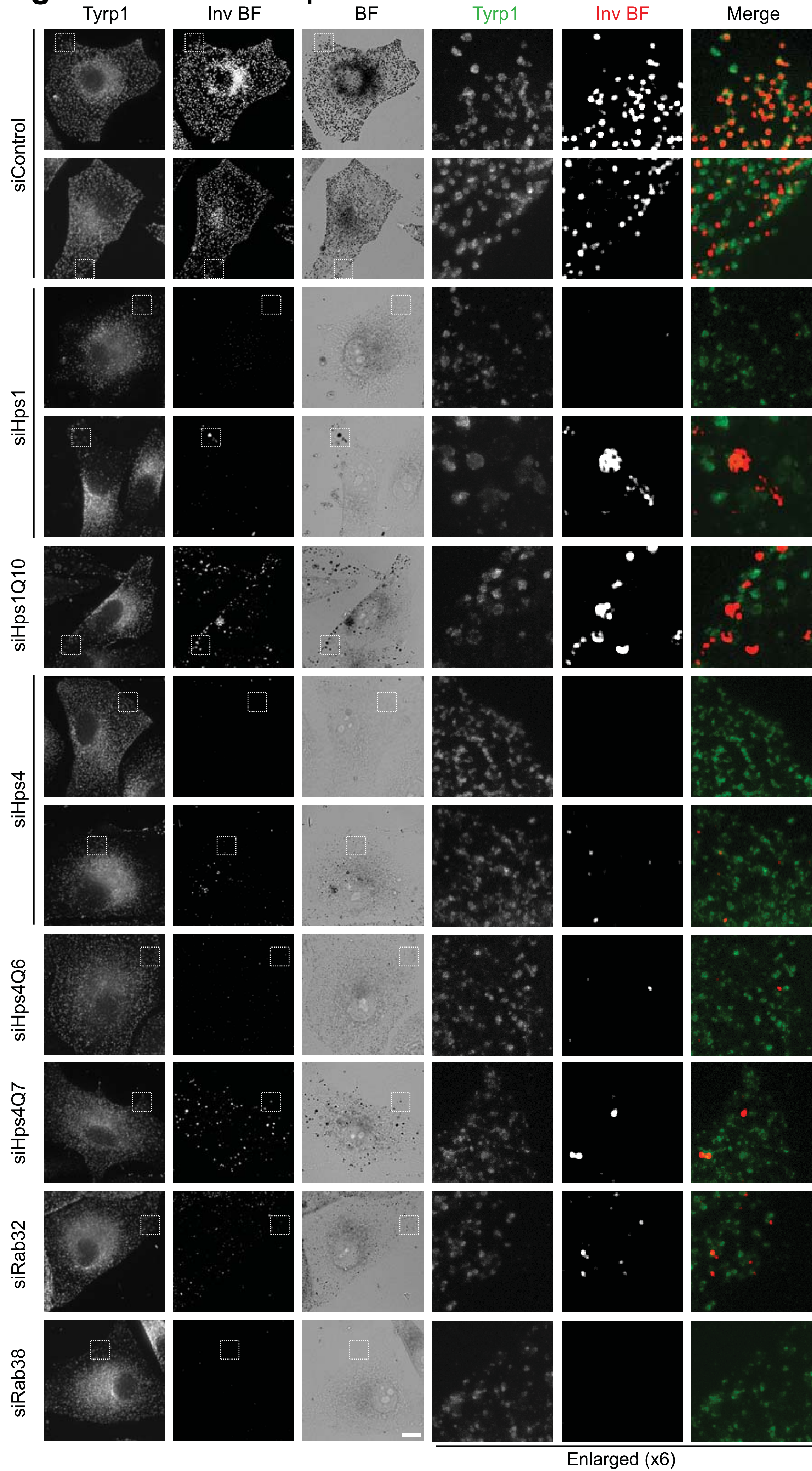

Figure S4. Melanosomes Are Lost from BLOC-3 Depleted Cells

MNT-1 cells treated with control, Hps1, Hps1Q10, Hps4, Hps4Q6, Hps4Q7, Rab32, or Rab38 siRNA duplexes for 8 days were PFA-glutaraldehyde fixed, and then stained for Tyrp1. A brightfield image (BF) was taken and inverted to more clearly show the dark melanosomes (Inv BF). Scale bar is 10 µm in non-enlarged panels.
